# Anxiety-related attentional characteristics and their relation to freezing of gait in people with Parkinson's: Cross-validation of the Adapted Gait Specific Attentional Profile (G-SAP)

**DOI:** 10.1177/1877718X251326266

**Published:** 2025-05-20

**Authors:** Uri Rosenblum, Adam J Cocks, Meriel Norris, Elmar Kal, William R Young

**Affiliations:** 1 Department of Health Sciences, College of Health, Medicine and Life Sciences, Brunel University London, UK; 2 Faculty of Health and Life Sciences, University of Exeter, Exeter, UK; 3 Centre for Clinical and Cognitive Neuroscience, Brunel University London, UK; 4 Division of Sport, Health and Exercise Sciences, College of Health, Medicine and Life Sciences, Brunel University London, UK

**Keywords:** gait specific attentional profile, freezing of gait, Parkinson's disease, confirmatory factor analysis, rumination, conscious movement processing, anxiety, physiological arousal

## Abstract

**Background:**

Anxiety often exacerbates freezing of gait (FOG) in people with Parkinson's (PwP). Anxiety-related attentional processes and associated processing inefficiencies, like conscious movement processing (CMP) and ruminations, can substantially impact movement control. However, their impact on FOG remains largely unexplored.

**Objective:**

To validate an adapted 10-item (1–5 Likert scale) Gait-Specific Attentional Profile (G-SAP) in PwP and assess if adapted G-SAP-subscales (Physiological Arousal, CMP, Rumination, and Processing Inefficiencies) are associated with self-reported FOG frequency.

**Methods:**

We recruited 440 PwP (M_age_ = 65.5 ± 8.7; 5.8 ± 5.0 years since diagnosis) across the UK. Participants completed the adapted G-SAP and questionnaires on demographics, medical background, and FOG frequency. We assessed adapted G-SAP's internal consistency, structural validity, and subscale scores associations with FOG frequency.

**Results:**

The adapted G-SAP showed acceptable internal consistency (α≥0.66) and acceptable/good model fit (comparative fit index = 0.976). Physiological Arousal and CMP subscale scores presented weaker correlations for PwP with FOG (PwP + FOG, r = 0.52) compared to PwP without FOG (PwP-FOG, r = 0.77; p = 0.006). Higher Rumination (OR: 1.323, 95%CI: [1.214–1.440]) and Physiological Arousal (OR: 1.195, 95%CI:[1.037–1.377]) were significantly associated with higher FOG frequency, controlling for age, time since diagnosis and balance/gait problems.

**Conclusions:**

The adapted G-SAP is reliable and convenient to measure and identify potentially maladaptive anxiety-related attentional processes that may impact FOG. Results suggest that PwP who experience more worrisome thoughts and greater physiological arousal in daily life are likelier to freeze. Compared to PwP-FOG, for PwP + FOG high physiological arousal was associated with reduced goal-directed focus of attention. Future research will determine if this is a causal risk factor.

## Plain language summary

Anxiety can worsen freezing of gait in people with Parkinson's. It often leads to worrisome thoughts and influences how people pay attention to their walking. We think that these changes in attention can substantially influence peoples’ movement – for better or worse. However, there is a lack of research on this topic, and reliable assessment tools are missing.

Therefore, we tested if we could assess changes in the thoughts and attention of people with Parkinson's, using a questionnaire (Gait-Specific Attentional Profile (adapted G-SAP)), previously used in older adults without Parkinson's. This questionnaire aims to measure people's perception of their physiological arousal (how anxious they feel), conscious movement (attention they direct to walking), rumination (self-preoccupation with worries about failure and expectations of negative consequences), and thinking efficiency (the ability to focus on different tasks). We also investigated if people who experience freezing show different attentional characteristics compared to people who do not experience freezing. Four-hundred and forty people with Parkinson's filled the adapted G-SAP questionnaire. We confirmed the questionnaire's reliability and found that people who indicated to have more worrisome thoughts and greater physiological arousal also experienced freezing more often. Our findings suggest that people with Parkinson's who experience higher rumination are more likely to experience freezing of gait. The adapted G-SAP represents a short and convenient tool for identifying potentially negative attentional and thinking processes that may increase freezing frequency. With further research to better understand the causes of freezing of gait and their relation to rumination and physiological arousal the adapted G-SAP could be used to monitor and test treatment effect on these constructs in the context of FOG frequency.

## Introduction

Freezing of gait (FOG) is a “brief episodic absence or marked reduction of forward progression of the feet despite the intention to walk”;^1^ a debilitating symptom of Parkinson's disease, with prevalence reported between ∼37% for early stage (≤5 years) and ∼64% for advanced stage (≥ 9 years)^2^ and as high as 80% in later stages.^2^ FOG is associated with increased prevalence of falls^3^ and reduced quality of life of People with Parkinson's (PwP).^4,5^ FOG can manifest across different so-called phenotypes, such as trembling in place, shuffling forward, and akinesia. While there currently is no broad consensus on the specific pathogenesis of FOG, common triggers have been identified, such as doorways/narrow spaces, turning and multi-tasking.^6,7^ One specific factor that is often implicated in exacerbating FOG frequency and duration is anxiety.^8^ It is suggested that increased anxiety (i.e., physiological arousal) overwhelms pre-existing defective limbic circuitry and noradrenergic pathways, leading to neural and cognitive inefficiencies that worsen freezing.^8,9^ Based on the Attention Control Theory (ACT),^10^ this paper aims to study the role of specific anxiety-related attentional characteristics in FOG, as this may ultimately help inform intervention development.

ACT^10^ describes how anxiety may lead to preferential engagement of the ventral stream of information processing and associated stimulus-driven attentional system at the expense of a decreased influence of the goal-directed (dorsal) attentional system.^10^ According to these predictions, PwP with high arousal would allocate attentional resources to processing threat-related stimuli (e.g., worrisome thoughts related to freezing, or threatening task-irrelevant distractors, like doorways) rather than to goal-directed attentional processes (e.g., focusing on the intended step), resulting in more frequent and severe freezing. However, based on ACT, and its predecessor Processing Efficiency Theory,^11^ it is also predicted that such negative effects of anxiety could be offset if PwP manage to maintain their attentional focus toward the intended movement goal, through increased mental effort and/or inhibition of distraction by threat-related stimuli. These theories are well supported by empirical evidence in performance contexts, such as sport or surgery^12–14^ as well as functional gait, especially in older adults at risk of falling.^15^ However, relatively little is known about the role of these proposed anxiety-related attentional characteristics in the context of FOG. There is evidence that worrisome thoughts and rumination (i.e., self-preoccupation with concerns over failure and expectations of negative consequences^16^) are triggered by stressful situations and are prevalent in individuals high in trait anxiety^17^ such as older adults fearful about falling. While such worries may be acted on to make adaptive changes to behavior (e.g., walking with an assistive device), processing such thoughts while walking will be cognitively demanding, and will bias an individual's attention toward potential threats to balance^18,19^ (or in PwP: toward potential triggers for FOG). This may distract attention away from the movement task at hand and thus exacerbate FOG. These assertions are particularly relevant in the context of Parkinson's where increased conscious monitoring of ongoing movements is required to compensate for loss of movement automaticity.^20^

The negative effects of anxiety-induced rumination and worry may therefore be minimized by investing more conscious attention into controlling and monitoring ongoing movement. Nonnekes and colleagues^21^ identified 59 unique strategies for improving mobility in PwP, “where an overarching working mechanism involved in all was allocation of attention to gait, the introduction of goal directedness, and the use of motor programs that are less automatized than those used for normal walking”.^21^ Further, cueing and movement strategy (e.g., weight-shifting) interventions for FOG are thought to be effective, at least in part, because they compensate for deficient automaticity by engaging cortical networks involved in goal-directed attention.^22,23^ However, conscious goal-directed strategies are effortful and cognitively demanding and will become more difficult to employ as anxiety and overall task demands increase.^10^ This may be especially true for PwP, who often already demonstrate both strong conscious control of movement,^24^ as well as deficits in executive functions, such as inhibition^25–29^ and shifting of attention.^30–36^ This will likely compromise their ability to block out worrisome thoughts and shift attention back toward the movement task at hand.

There are different tools available that allow us to quantify the degree to which people consciously control their movements. One of the most widely used is the Movement Specific Reinvestment Scale (MSRS),^37^ a 10-item questionnaire that consists of “Movement Self-Consciousness” (5 items) and “Conscious Motor Processing” (5 items) subscales. Although the MSRS is widely used in movement science, multi-level meta-analysis has shown that MSRS scores are not significantly associated with motor performance^38^ Furthermore, key limitations of the MSRS are that it is not specific to gait and balance (but rather to movements in general), and that it does not allow us to distinguish between different attentional changes that are likely to co-occur with increased CMP, which could impact on gait and balance as discussed earlier (i.e., rumination, physiological arousal and processing efficiency). For instance, Young et al.^18^ found that people may often misinterpret the MSRS and report higher scores as a reflection of worrisome thoughts (i.e., ruminations) rather than CMP specifically.

To address the above issues, Young and colleagues^18^ developed the Gait-Specific Attentional Profile (G-SAP). This is a short self-report instrument that was specifically developed for use in older adults with balance impairments. The instrument allows the measurement of the degree to which individuals experience heightened somatic anxiety (Physiological Arousal subscale, note: the subscale does not fully account for the perception of physiological arousal as expressed by skin conduction or heart rate for example), conscious attention to movement (Conscious Movement Processing, CMP subscale), worrisome thoughts (Rumination subscale), and processing inefficiencies (PI subscale) when *walking* in daily life.^18^ Young et al.,^18^ showed that “unlike the MSRS, the G-SAP subscale of CMP significantly predicted several gait characteristics including velocity (*p *= 0.033), step length (*p *= 0.032), and double limb support (*p *= 0.015).“ They concluded that The G-SAP allows the distinct measurement of these specific attentional factors, and specifically in relation to gait, addressing the issues with the MSRS outlined above. When considering the specific items in the adapted G-SAP, we argue that high scores will predominantly reflect negative valence on the whole, and that higher scores on physiological arousal subscale are indicative of physical stress and tension, while the CMP subscale is likely to capture motivation and focus in relation to the task at hand. Nonetheless, it is important to acknowledge that, like any self-reported outcome measure, the adapted G-SAP scale will also inherently have limitations such as an individual's fluctuations in awareness and recall bias.

Furthermore, literature is emerging in support of factoring in the negative effects of anxiety-induced rumination and worry on PwP, the G-SAP could provide insight into the role of these different constructs in the context of FOG in this population. Indeed, a recent study by Cockx et al.^39^ used the G-SAP to explore potential relationships between the above constructs and the propensity to experience FOG when navigating doorways. They found that people who show freezing in response to doorways have significantly longer disease duration and higher scores for all G-SAP subscales compared to those who do not freeze in response to doorways. While this report does not identify freezing pathology as an independent factor associated with higher G-SAP scores, the results clearly emphasize the potential of utilizing self-reported attentional processes to deepen our understanding of the relationship between anxiety and FOG in different contexts. Further, a limitation of Cockx et al.^39^ is that no comprehensive cross-validation has yet been conducted to demonstrate the reliability and validity of the GSAP for PwP with (PwP + FOG) and without FOG (PwP-FOG). Lastly, the original G-SAP questionnaire consists of 11 questions across the same four domains. However, item A2, from the ‘Physiological Arousal’ sub-scale, does not fully capture/align with the subscale of Physiological Arousal. That is, item A2 required respondents to indicate to what extent they are concerned about other people's thoughts about them. While this item was allocated to the more broadly named ‘anxiety’ sub-scale in the original G-SAP validation (in a heterogeneous group of older adults), there is a concern that this concept does not fit with the more specific construct that better-reflects physiological arousal and may reflect aspects associated with *cognitive* anxiety (i.e., rumination).

Therefore, this study aimed to (1) validate an adapted version of the G-SAP scale for use in PwP + FOG and PwP-FOG (adapted G-SAP), where item A2 in the original G-SAP was removed from the ‘Physiological Arousal’ sub-scale. This has the benefit of creating a clearer contrast between Physiological Arousal (A1, A10) and Rumination (A3, A4, A6); and (2) determine if self-reported FOG frequency in daily life is independently associated with different adapted G-SAP sub-scales. In line with the literature above,^17,39^ we hypothesized that more frequent FOG would be associated with higher scores on all subscales but would show strongest unique associations with Rumination subscale scores.

## Methods

### Participants

Four hundred and forty PwP were recruited through advertisements across the Parkinson's UK network (circulated through email across the UK) as well as in-person invitations at local Parkinson's support groups in West London. The advertisements included a link to an online survey. No incentives were provided for participation.

Participants were eligible for inclusion if they had a diagnosis of Parkinson's, it was explicitly stated within the survey, that people should only respond if they have a formal diagnosis of Parkinson's and no other diagnoses and had sufficient command of the English language to understand and complete the survey. Participant characteristics are presented in Table 1.

**Table 1.** Characteristics of patients with (‘PwP + FOG’) and without (‘PwP-FOG’) freezing of gait.

	PwP-FOG (N = 236)	PwP + FOG (N = 199)		
	*“Never”* *(N = 236)*	*“Hardly ever” (N = 109)*	*“Most weeks” (N = 39)*	*“Every day”* *(N = 51)*
**Age in years**	65.9 ± 8.2 [43–81]^c^	63.3 ± 8.0 [40–81]^e^	64.3 ± 10.9 [41–85]^b^	68.7 ± 9.0 [46–86]^a^
**Any other diagnosis/condition^#^**				
*None*	186 (79%)	75 (69%)	30 (77%)	35 (69%)
*Arthritis*	16 (7%)	10 (9%)	3 (8%)	6 (12%)
*Neurological*	7 (3%)	6(5%)	2 (5%)	1 (2%)
*Back pain/problem*	9 (4%)	6 (5%)	1 (3%)	2 (4%)
*Other orthopedic*	12 (5%)	5 (5%)	1 (3%)	2 (4%)
*Cardiovascular*	2 (1%)	2 (2%)	0 (0%)	4 (8%)
*Other (e.g., diabetes, colitis, tendon injury)*	2 (1%)	5 (10%)	1 (3%)	0 (0%)
**Years since diagnosis**	4.3 ± 3.4*** [0–19]^a^	5.8 ± 4.6 [0–23]^d^	8.2 ± 5.8 [0–25]^a^	10.5 ± 7.1 [0–27]
**Tremor** *Not at all (1) - Very much so (5)*	2 (1) [1–5]^a^	2 (1) [1–5]	2 (2) [1–4]^a^	2 (2) [1–5]
**Problems with balance and gait** *Not at all (1) - Very much so (5)*	2 (1)*** [1–5]	2 (2) [1–5]	3 (2) [1–5]^b^	4 (2) [1–5]
**adapted G-SAP Sub-scale Scores**				
Physiological Arousal	5.0 ± 1.9*** [2–10]	6.4 ± 2.0 [2–10]	6.9 ± 1.9 [3–10]	7.4 ± 1.8 [4–10]
CMP	9.5 ± 3.0*** [3–15]	10.7 ± 2.6 [4–15]	11.1 ± 2.5 [5–15]	11.9 ± 2.2 [7–15]
Rumination	5.6 ± 2.5*** [3–13]	7.8 ± 2.8 [3–15]	8.4 ± 2.7 [3–13]	10.8 ± 2.8 [3–15]
PI	3.8 ± 1.7*** [2–9]	5.0 ± 1.7 [2–9]	5.2 ± 2.0 [2–9]	5.8 ± 2.1 [2–10]

**NB:** Continuous variables are expressed as mean ± standard deviation [range], while categorical variables are expressed as median (interquartile range) [range]; ^#^Note that some patients reported more than one additional condition, and that percentages will therefore add up to more than 100%; *** significant difference between PwP + FOG and PwP-FOG p < 0.001;

a – one missing value; b – two missing values; c – four missing values; d – five missing values; e – eight missing values

CMP: Conscious Movement Processing; PI: Processing Inefficiency

Institutional ethical approval was obtained from the College of Health, Medicine and Life Sciences Research Ethics Committee of Brunel University London (REF: 6473-MHR-May/2017- 7263-2). All participants provided online written informed consent prior to participation.

### Procedure

The online survey was hosted online using JISC Online Surveys (Bristol, UK). First, participants completed the online informed consent form, prior to completing several questions on their background. These included age in years, co-morbid diagnoses (e.g., orthopedic, neurological conditions), years since Parkinson's diagnosis, information on other Parkinson's symptoms such as tremor and balance problems; rated on 1 (“not at all”) to 5 (“very much so”) Likert scale), and self-reported FOG frequency (“never”, “hardly ever”, “most weeks”, “every day”, this item was not restricted to a specific time period). For the purpose of later analysis (see below), participants who reported to “never” freeze were categorized as PwP-FOG, while all others reporting higher scores were classified as PwP + FOG. To ensure that questionnaires were answered once by each participant we visually checked for duplicate scores.

### Adapted G-SAP questionnaire

Next participants completed an adapted G-SAP questionnaire (see Methods section in the Supplemental Materials). This adapted G-SAP consists of 10 questions across 4 different domains: Physiological Arousal (2 items), CMP (3 items), Rumination (3 items), PI (2 items). For each item, respondents indicate their level of agreement on a scale from 1 (“Not at all”) – 5 (“Very much so”). For each subscale, scores are then summed to create an overall subscale score.

### Data analysis and statistics

All data were analyzed with R version 4.2.2., unless stated otherwise. Alpha was set at 0.05.

#### Comparison of characteristics between PwP + FOG and PwP-FOG

Patient characteristics were presented using appropriate measures of central tendency and dispersion and compared between PwP + FOG and PwP-FOG using parametric and/or non-parametric tests as appropriate. Cohen's d was used as measure of effect size^40^ (‘cohensD’ function, ‘lsr’ package in R).

#### Validity and reliability of Gait-Specific Attentional Profile

We conducted confirmatory factor analysis (maximum likelihood estimation, CFA) to assess the adapted G-SAP's structural validity (AMOS, version 26; IBM, Chicago, IL). Specifically, we aimed to determine whether the data would fit the four-factor structure reported in the initial G-SAP validation study in healthy older adults.^18^ Pairs of error terms for items loading on same subscale/factor were allowed to co-vary if this improved model fit.

We present the overall model including standardized item-factor loadings, along with the following model fit tests: Chi-square statistics, both absolute (χ^2^; non-significant χ^2^indicates acceptable fit) and divided by degrees of freedom (χ^2^/df; values < 3 indicate acceptable fit); goodness-of-fit and comparative fit indices (GFI and CFI; values > 0.90 indicate acceptable fit, values > 0.95 indicate good fit); standardized root mean squared residual (SRMR; values < 0.08 indicate good fit); and root mean square error of approximation (RMSEA; values < 0.05 indicate good fit, values < 0.08 indicate acceptable fit).^41–43^

Next, we performed measurement invariance tests to determine if the adapted G-SAP's factor structure is similar for PwP + FOG and PwP-FOG. This consisted of three different steps. First, model fit was assessed when item-factor loadings were free to differ between PwP + FOG and PwP-FOG (configural invariance), subsequently with item-factor loadings fixed across these two groups (metric invariance), and finally with both the item-factor loadings and intercepts fixed (scalar invariance). Factor structure is considered similar if model fit remains similar, i.e., non-significant Δχ^2^ values, ΔCFI & ΔRMSEA < 0.010, ΔSRMR < 0.015.^44^

Finally, internal consistency was determined for each separate subscale/factor of the adapted G-SAP using ‘alpha’ function in the ‘psych’ package in R. Internal consistency was considered to be sufficient if alpha ≥ 0.65.^45,46^

#### Relation between Gait-Specific Attentional Profile and freezing of gait

Two-Way ANOVA (‘anova’ function, ‘car’ package in R) was used to compare adapted G-SAP scores between groups of patients with different freezing frequency. The different subscale scores served as the dependent variable and the independent variables were the adapted G-SAP subscales, freezing frequency, and their interaction (adapted G-SAP subscale X freezing frequency). For this analysis, adapted G-SAP scores were Z-transformed to allow for comparison between subscales with different score range. Post-hoc Tukey Honest Significant Difference was used to correct for multiple comparisons. Eta squared (η^2; ‘^etaSquared’ function in the ‘lsr’ package in R) was presented as measure of effect sizes.

Subsequently, we performed ordinal regression analysis to analyze the association between the different adapted G-SAP subscale scores and frequency of freezing (logit link function). Participant characteristics (i.e., age, years since diagnosis and experiencing balance problems) were controlled for as covariates in the model.

We then explored cutoff values for (i) PwP + FOG vs. PwP-FOG status and (ii) freezing everyday vs. freezing less frequently (combined freezing “hardly ever” and “most days”). This exploratory phase was pursued to provide some clinical meaning for questionnaire scores. We selected the adapted G-SAP subscale score with the highest (significant) odds ratio in the ordinal regression to further explore diagnostic accuracy. Specifically, we used area under the curve (AUC) analysis (SPSS version 29; IBM, Chicago, IL) to explore cut-off scores based on optimal sensitivity vs. specificity trade-off (Youden's index^47^). In the context of a ROC curve analysis the index represents the optimal cutoff point on the curve which is the furthest from the identity line (i.e., chance level).

#### Sample size calculation

We aimed for an overall sample of ∼400 participants, which is the recommended sample size for factor analysis involving 3–6 factors (subscales), 3 items per factor, and conservative expected factor loadings of 0.4.^48^ This sample size was also anticipated to result in at least 30 participants for each freezing frequency category, as required for the 2-way-ANOVA and the ordinal regression model.^49,50^

## Results

### Participants

In total, 440 patients with Parkinson's disease completed the questionnaire. Five were excluded from the CFA due to missing freezing data, leaving 435 patients. Twenty-seven patients were excluded only from the regression analysis, because of missing data regarding freezing (N = 5), years since diagnosis (N = 9), and/or age (N = 16). Thus, regression analysis was performed on the remaining 413 patients. Detailed patient characteristics can be found in Table 1.

### PwP + FOG vs. PwP-FOG

In all, 236 patients reported that they never experience freezing of gait (PwP-FOG), while 199 patients reported to experience freezing (PwP + FOG). On the whole, PwP + FOG were of similar age as PwP-FOG (64.9 ± 9.3 vs. 65.9 ± 8.2, respectively; t_(377.78)_ = 1.11, Cohen's d = 0.11, p = 0.867). However, PwP + FOG had longer time since diagnosis of Parkinson's disease (7.49 ± 5.91 vs. 4.3 ± 3.4, respectively; t_(295.05)_ = −6.63, Cohen's d = 0.67, p < 0.001), more self-reported balance problems (Mdn = 3, IQR = 2 vs. Mdn = 2, IQR = 1, respectively; W = 11,841, Cohen's d = 0.95, p < 0.001). However, presence of tremor complaints (Mdn = 2, IQR = 2, vs. Mdn = 2, IQR = 1; W = 23,639, Cohen's d = 0.00, p = 0.619) and of additional medical conditions (30% vs.21%; χ2_(1)_ = 2.253, Cohen's d = 0.20, p = 0.133) were similar for PwP + FOG and PwP-FOG, respectively.

### Gait-specific attentional profile validation for PwP

#### Structural validity: confirmatory factor analysis (CFA)

**Figure 1.**
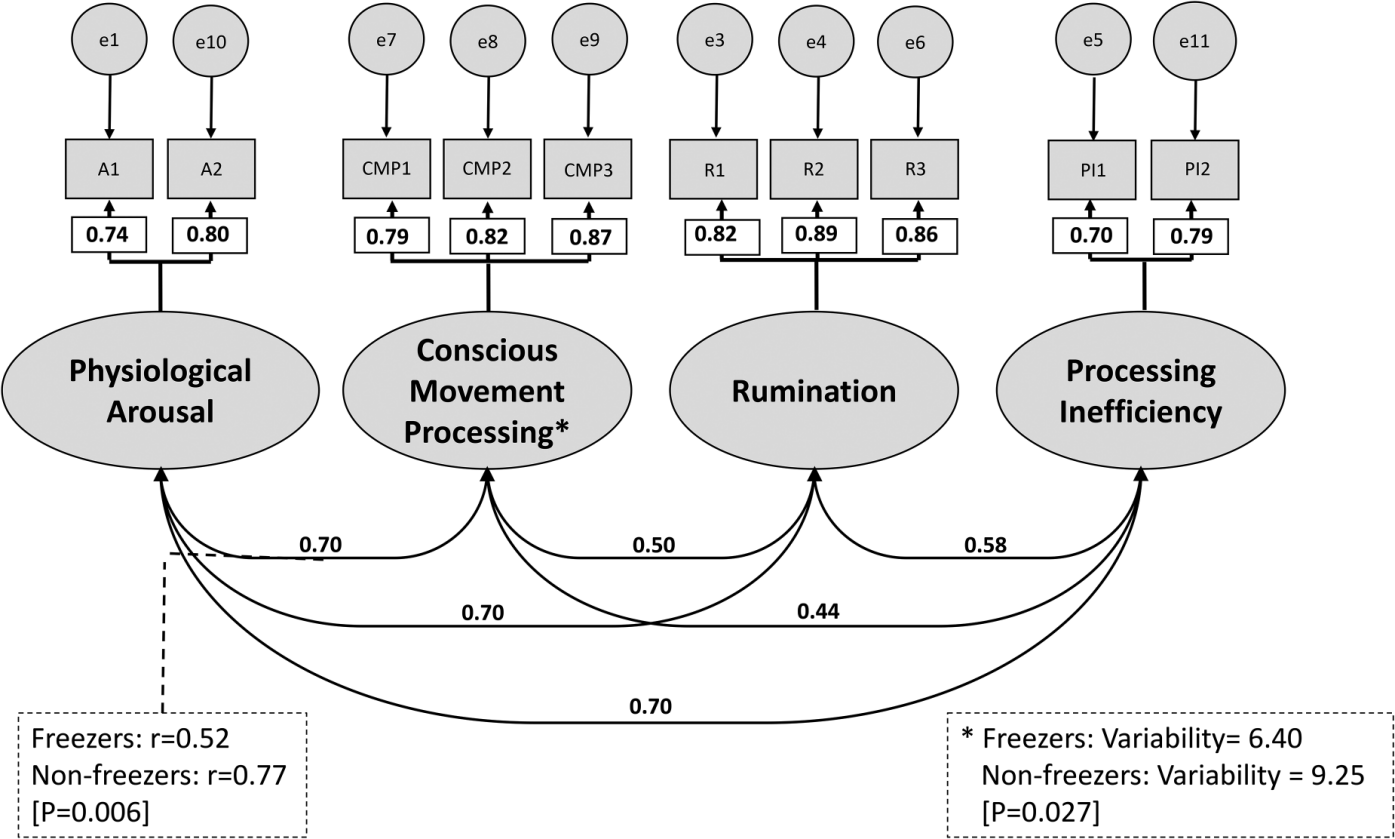
Final overall configural model yielded by the confirmatory factor analysis. Shown are the standardized item-factor loadings and the covariances between the latent factors for all participants combined and specifications of significant differences between people with Parkinson's with (PwP + FOG) and without freezing of gait (PwP-FOG) in an adjusted partial scalar model. Dotted lines indicate that covariances are significantly different between PwP + FOG and PwP-FOG, with the respective values for both groups. *indicates significant variability differences between PwP + FOG and PwP-FOG, with the respective values for both groups. Abbreviated item numbers refer to the respective items for each latent factor on the Adapted Gait Specific Attentional Profile (adapted G-SAP, see Supplemental Material). NB: PA: Physiological Arousal; CMP: Conscious movement processing; PI: Processing inefficiency; R: Rumination; e: residual error. See Supplemental Table 1 for details on final model selection.

The overall CFA model is presented in Figure 1. Medium to strong correlations were observed between all factors, and especially between the factor of ‘Physiological Arousal’ and ‘Conscious Movement Processing’ (r = 0.700), Rumination (r = 0.700) and Processing inefficiency (r = 0.700), respectively. Standardized item-factor loadings were all positive and high (≥0.70). Model fit indices were acceptable to good (χ^2^_(29)_ = 78.591, *p *< 0.001; χ^2^/df = 2.710; CFI = 0.978; GFI = 0.965; SRMR = 0.034, RMSEA = 0.063[0.046, 0.080]).

The model demonstrated configural and metric measurement invariance (Supplemental Table 1). Scalar measurement invariance was borderline satisfactory. Further analysis using backward releasing of constraints revealed that this was primarily due to a significant between-group difference in covariance between the subscales ‘Physiological Arousal’ and ‘CMP’ (*r*_PwP + FOG _= 0.52, *r*_PwP−FOG_ = 0.77, respectively; *Z *= −2.776, *p *= 0.006, see Supplemental Figure 1), and to a lesser extent to reduced variability in ‘CMP’ subscale scores among PwP + FOG compared to PwP-FOG (6.40 and 9.25, respectively; *Z *= −2.205, p = 0.027). Unconstrained values for the ‘Physiological Arousal’ and ‘CMP’ covariance, and ‘CMP’ variance led to acceptable partial scalar invariance (Supplemental Table 1).

Overall, the CFA therefore confirmed the hypothesized four-factor structure of the adapted G-SAP and demonstrated that the scale is suitable to compare scores between PwP + FOG and PwP-FOG.

#### Internal consistency

Internal consistency of the adapted G-SAP was confirmed. For the sample overall, standardized Cronbach's alpha values were 0.75, 0.86, 0.89 and 0.67 for Physiological Arousal, CMP, Rumination and PI, respectively. For PwP + FOG the respective standardized Cronbach's alpha values were 0.69, 0.82, 0.86, and 0.65, while for PwP-FOG these were 0.70, 0.87, 0.85, and 0.61.

### Adapted G-SAP scores: differences between groups

**Figure 2.**
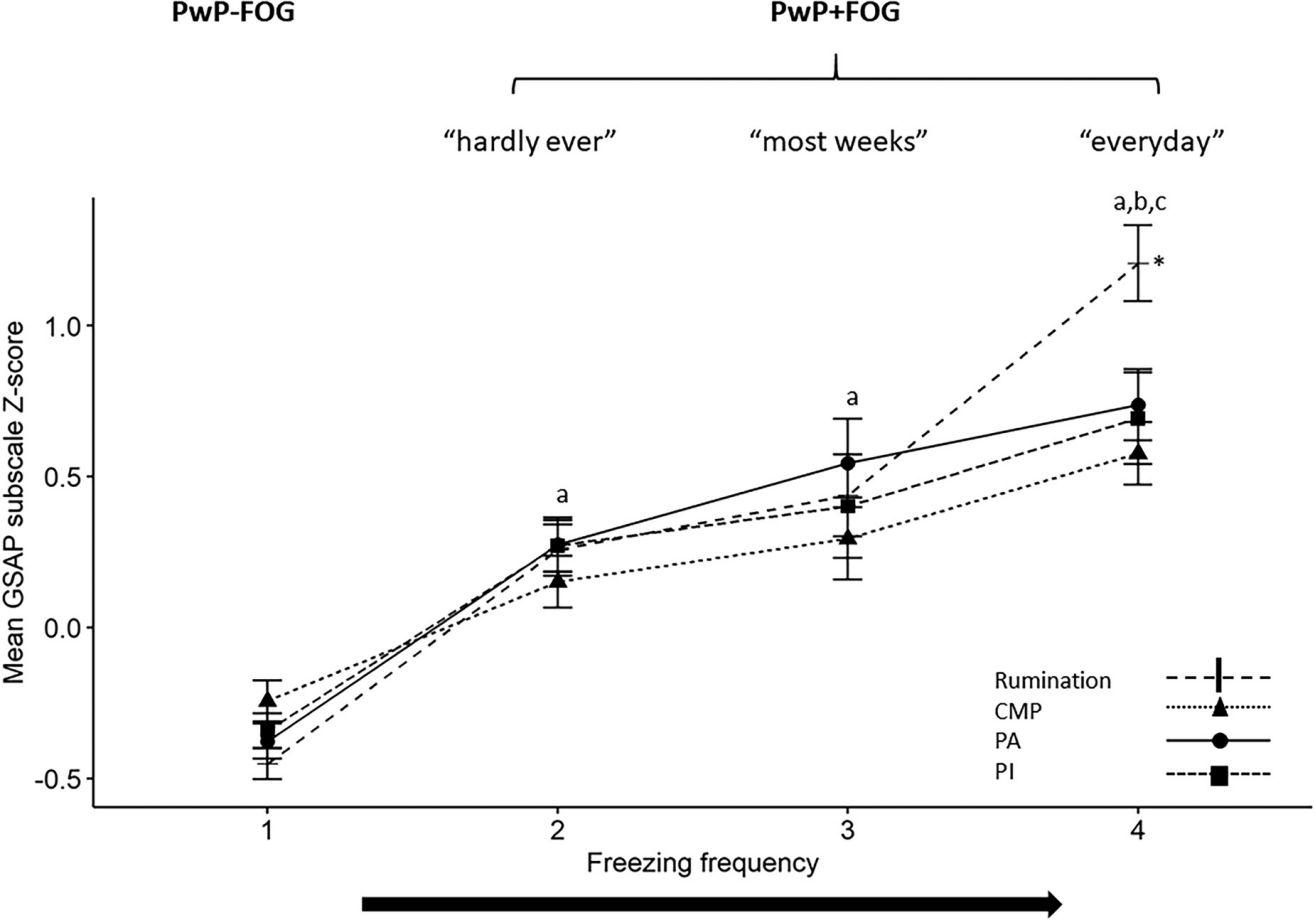
Mean (± standard error) Z-scores for each of the four subscales of the Gait-Specific Attentional Profile. Data are presented separately as a function of self-reported frequency of freezing of gait.

Note: homogeneity of variances was assessed by plotting the residuals against the fitted values. Since there was no apparent relationship between the two, homogeneity was assumed. However, Levene's test and Shapiro-Wilk test were significant (p = 0.008 and p < 0.001, respectively), hence not supporting homogeneity and normality assumptions (see Supplemental Figure 2 for details). This should be considered when interpreting the results.

Two-way ANOVA, with ANOVA type III sum of squares analysis for unbalanced design (i.e., unequal number of participants in each group), was implemented to explore the differences in adapted G-SAP subscale scores between PwP + FOG and PwP-FOG. We found a significant main effect of freezing frequency (F_[3]_ = 122.43, η^2 ^= 0.17, p < 0.001) and a significant subscale X freezing frequency interaction (F_[9]_ = 2.63, η^2 ^= 0.01, p = 0.005), but no main effect of adapted G-SAP subscale (p = 0.178). Post-hoc tests (Tukey) showed that across subscales, transformed adapted G-SAP scores were significantly higher for each subgroup of PwP + FOG compared to patients who never experience freezing, with highest scores for freezes everyday group (mean overall Z-scores: PwP-FOG −0.353 ± 0.91, freezes hardly ever 0.24 ± 0.90, freezes most weeks 0.42 ± 0.92, and freezes everyday 0.80 ± 0.93, p < 0.001). Furthermore, we found no differences between subscale scores within each freezing frequency subgroup (p > 0.39), except for significantly higher Rumination compared to conscious movement processing in the freezing everyday group (1.21 ± 0.90 vs. 0.58 ± 0.75, p = 0.039). Results are summarized in Figure 2.

a- significantly greater overall score than PwP-FOG group, p < 0.001

b- significantly greater overall score than freezing ‘hardly ever’ group, p < 0.001

c- significantly greater overall score than freezing ‘most weeks’ group, p < 0.001

* Significantly different from CMP, p = 0.039

CMP: Conscious movement processing; PA: Physiological Arousal; PI: Processing Inefficiency.

### Adapted G-SAP scores: association with frequency of freezing

Ordinal regression results are presented in Table 2. Twenty-seven participants with missing responses for age, years since diagnosis or experiencing balance problems, were not included in the regression analysis. Of the adapted G-SAP subscales, only higher Rumination subscale scores (OR = 1.323, 95% CI = [1.214, 1.440]) and higher Physiological Arousal subscale scores (OR = 1.195, 95% CI = [1.037, 1.377]) were associated with significantly greater odds of experiencing more frequent freezing. With regard to the control variables, only years since diagnosis was significantly associated with freezing frequency (OR = 1.140, 95% CI = [1.091, 1.192]).

**Table 2.** Results of ordinal regression analysis of adapted G-SAP scores as a function of freezing of gait frequency.^a^[Table-fn table-fn5-1877718X251326266]

	OR [95% CI]	Wald χ^2^ (df = 1)	*p*
Age in years	0.988 [0.964, 1.012]	0.942	0.332
Years since diagnosis	1.140 [1.091, 1.192]	33.329	**<0**.**001**
Processing Inefficiency	1.124 [0.991, 1.274]	3.329	0.068
Physiological Arousal	1.195 [1.037, 1.377]	6.005	**0**.**014**
Rumination	1.323 [1.214, 1.440]	40.944	**<0**.**001**
Conscious Movement Processing	0.961 [0.873, 1.056]	0.684	0.408
Balance/gait problems^b^	0.559 [0.308, 1.014]	3.669	0.055

NB: OR = odds ratio, values > 1 indicate increase in odds of experiencing more frequently freezing; df = degrees of freedom; Model-parameters: Improvement in fit vs. intercept-only model (*χ*^2 ^= 201.903, df = 7, *p *< 0.001); Goodness-of-fit indices: Pearson (*χ*^2 ^= 1120.421, df = 1217, *p *= 0.977), Deviance (*χ*^2 ^= 720.290, df = 1217, *p *= 1.000); Nagelkerke pseudo R^2 ^= 0.435.

^a^ The assumption of lack of multicollinearity was met (all VIFs 1.063–2.130), but the proportional odds assumption was not, as evidenced by a significant test of parallel lines (χ2 = 31.887, df = 14, p = 0.004). Hence, we fitted a less restrictive model (i.e., multinomial logit model) which fit a model for every level of the freezing frequency by itself, see summary in Supplemental Table 2. We did not find significant differences between the models.

^b^ Reference category is group with self-reported problems with balance or gait (N = 293). Significant *p* values are maked bold.

### Adapted G-SAP scores cut off for associations with freezing frequency

**Figure 3.**
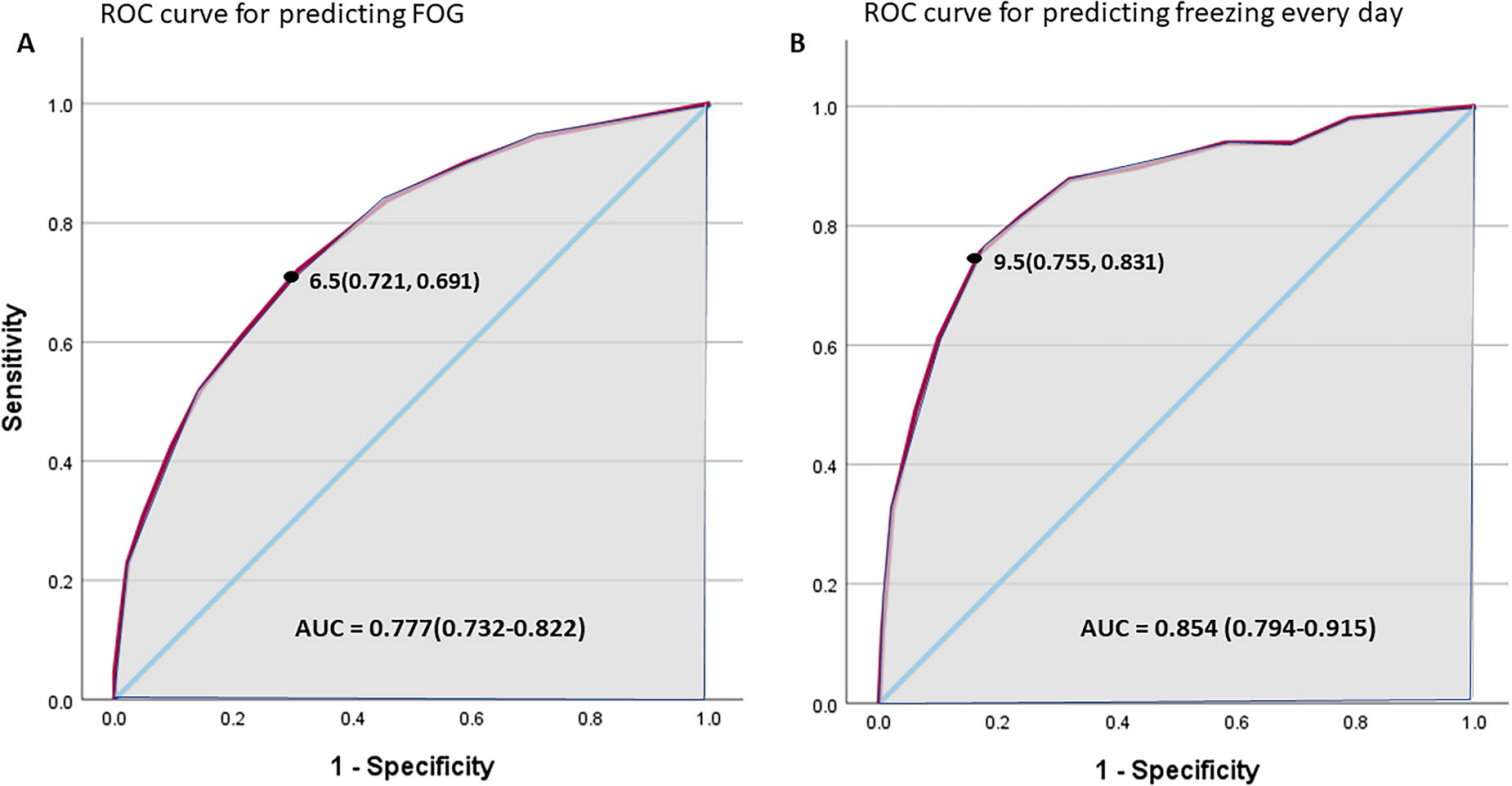
Receiver Operating Characteristic (ROC) curve (solid line) for predicting FOG (A) or freezing every day (B) using the adapted G-SAP, rumination sub-scale. Diagonal line represents a random prediction. Grey area represents the area under curve (AUC) and black point represents the cut-off point with sensitivity and specificity in parenthesis.

As Rumination had the largest effect size for the difference between PwP + FOG and PwP-FOG and highest odds ratio in the ordinal regression, and as ruminations are more likely to be negative (task-irrelevant) per se while performing a task, this sub-scale was used to explore cutoff values in the ROC analysis. Figure 3 presents the ROC curve for predicting FOG (Figure 3A) and freezing every day (Figure 3B). AUC was 0.777 (sensitivity 0.721 and specificity 0.691) and 0.854 (sensitivity 0.755 and specificity 0.831), for distinguishing PwP-FOG from PwP + FOG and freezing every day, respectively, indicating good diagnostic accuracy. Rumination subscale cut-offs scores of 6.5 and 9.5 were found to be the optimal cut-offs (i.e., highest Youden's index) for classifying PwP + FOG vs. PwP-FOG and for freezing ‘every day’, respectively (Supplemental Table 3). These might serve as initial cut-off points that can help identify PwP for whom worries (i.e., ruminations) are so high that they may become useful targets for intervention.

## Discussion

This study supports the validity and internal consistency of the adapted G-SAP to measure attentional factors (Physiological Arousal, Rumination, CMP and Processing Inefficiencies) implicated in influencing FOG in PwP. Overall, confirmatory factor analysis and measurement invariance testing showed that the adapted G-SAP has a four-factor structure with similar constructs to the original scale validated in community-dwelling older adults,^18^ with each of these subscales also showing sufficient internal consistency. Given that current evidence points to a clear causal influence of anxiety on FOG, we suggest that the adapted G-SAP sub-scale scores offers a method to quantify anxiety-related attentional processes implicated in this relationship. However, causality cannot be inferred from these data. Finally, ordinal regression revealed significant associations between FOG frequency and both Physiological Arousal and Rumination adapted G-SAP subscales, with strongest associations reported for the latter.

Findings of lower covariance between Physiological Arousal and CMP in PwP + FOG compared to PwP-FOG, suggest that PwP + FOG have reduced ability to utilize CMP to overcome adverse effects of physiological arousal. CMP is presumably employed as a mechanism to compensate for defective automaticity associated with Parkinson's disease. Likewise, compensatory cognitive processes are likely to be engaged in an attempt to mitigate any adverse influence of arousal on motor performance. Similar CMP values between PwP-FOG and PwP + FOG supports the interpretation that this relationship is not specific to FOG pathology.

Significantly higher Rumination scores compared to CMP scores among PwP with the most frequent freezing (i.e., on a daily basis), suggests that rumination might be a disrupting factor to utilizing CMP. When considering these results together, we propose that Rumination contributes to freezing through disruption of conscious goal-directed behavior that typically is required to maintain motor performance among PwP. We contend that these more frequent ruminations are likely to exacerbate FOG though the disruption to goal-directed focus while performing a task. However, increased self-reported ruminations in PwP + FOG group are also likely to be partially driven by previous experiences of movement difficulties as well as deficits in cognition and associated top-down control. Together, these interpretations form the basis for a potential bi-directional relationship that serves to increase scores in the Rumination sub-scale in PwP + FOG. However, it is also important to interpret the stronger covariance between arousal and ruminations in PwP + FOG compared to PwP-FOG, with reference to this bi-directional relationship. Walkers are likely to engage in worrisome thoughts in situations where their balance is threatened and recent work in healthy older adults suggests that the worrisome thoughts can be the product of a negative interpretation of bodily responses (i.e., perceived arousal/stress).^51^ We also speculate that the development and maintenance of worrisome thoughts might be exacerbated in individuals with increased autonomic dysfunction. We explored cut-offs for scores on the Rumination subscale to distinguish PwP-FOG from PwP + FOG (cut-off: 6.5), and to distinguish those with most frequent freezing from those with less frequent freezing (cut-off: 9.5). These results should be interpreted with caution, as sensitivity and specificity were between 0.7 and 0.8 leaving a large margin for type 1 error.

### Adapted G-SAP reliability

The G-SAP scale was originally developed and validated in healthy older adults with the four attentional subscales selected due to their implication in influencing the control of balance and gait and therefore fall-risk.^18^ Our results support the reliability of the adapted G-SAP to measure these same constructs in PwP (Physiological Arousal, Rumination, CMP and Processing Inefficiencies). It is important to note that researchers using the original G-SAP scale (https://osf.io/n7rcm/) to evaluate PwP, can adapt outcomes to the adapted version presented here (i.e., adapted G-SAP), by simply removing item A2 from the subscale of Physiological Arousal (formerly labelled as ‘Anxiety’). For results of the original GSAP reliability, including item A2, see the Supplemental Material.

### Associations between FOG severity and attention related cognitive processes

ACT describes how anxiety may shift cognitive processes away from goal-directed (dorsal) attentional system to information processing and associated stimulus-driven attentional system.^10^ This phenomenon is apparent in PwP and highlighted in our findings.

#### Rumination

Researchers have previously demonstrated a relationship between physiological arousal and FOG,^52^ typically inferred through observations of altered heart rate and/or skin conductance around FOG onset.^52,53^ This relationship is unlikely to be linear. Indeed, traditional conceptualizations of the relationship between arousal and motor performance (e.g., Yerkes-Dodson's Law^54^) strongly implicate that an optimum level of arousal exists for a given task and that this might fluctuate based on several factors, such as individual characteristics of the performer. This notion has already been proposed in the specific context of FOG.^55^ It infers that people may require a certain level of arousal to allocate attention in a manner that is sufficient to compensate for deficient automatic motor control processes. However, heightened arousal beyond this point is likely to compromise movement. Given existing literature^12–14,56^ and conceptualizations in older adults with concerns about falling,^18^ we suggest that emergence of cognitive anxieties (i.e., rumination) could be particularly problematic. The primary reason for this is that worrisome thoughts, while potentially related to adaptive decision-making around a given task (e.g., opting to use a walking stick or not), are irrelevant to current motor output and therefore likely to add unnecessary cognitive demands that exacerbate attention related cognitive processing inefficiencies. The cross-talk model suggests these may culminate in increasing limbic load (secondary to higher levels of anxiety and competing inputs) which in turn could provoke FOG by overloading the striatum, interfering with normal basal ganglia motor processing.^57^ As such, worries experienced in daily life could serve as distractions from consciously processing movements in much that same way as that observed in laboratory-based studies using cognitive dual-tasking paradigms that are known to exacerbate FOG.^32,58^ However, change in *physiological* arousal/*somatic* anxiety, as outlined by the ACT,^10^ is unlikely to be the sole anxiety-related variable that exacerbates FOG in daily life. Our data suggest that a marker of *cognitive* anxiety (namely worrisome thoughts as measured using the Rumination subscale) is substantially increased in people with frequent FOG and is the factor most strongly associated with FOG severity (independently from Physiological Arousal). Further study of the effects of rumination and worrisome thoughts of FOG prevalence and severity is warranted. Future work should perform cluster analyses on G-SAP sub-scale scores and explore associations with outcomes reflecting gait impairment and either adaptive or maladaptive cognitive processing.

#### Physiological arousal and CMP

In healthy populations performing skilled motor tasks, such as in the context of sport, military or surgery, conscious control of movement can compromise performance by virtue of an over-reliance on explicit knowledge and reduction in implicit/automatic control processes.^59^ However, the adaptive role of CMP has been demonstrated in people whose automaticity is compromised to some extent, such as older adults with reduced functional balance or stroke survivors.^60,61^ We argue that these observations might be extrapolated to the context of Parkinson's, where basal ganglia impairments cause pronounced deficiencies in automaticity, thereby increasing reliance on CMP as a necessary compensatory mechanism. Indeed, in the current study, we found evidence to suggest that PwP-FOG may have been better able to offset negative effects of arousal compared to PwP + FOG by engaging in increased CMP, as the PwP-FOG demonstrated greater covariance between Physiological Arousal and CMP. It might be the case that PwP + FOG are already highly engaged in CMP regardless of their anxiety level, potentially reflecting more progressed disease, and hence, greater deficiencies in automaticity that need to be compensated. This could potentially create a ceiling effect, where there is limited scope for investing greater CMP. This might be an explanation for the lower CMP and arousal covariance for the PwP + FOG.

### Anxiety management in the context of FOG

The understanding of the relationship between anxiety and FOG in recent years along with growing awareness of anxiety's detrimental effect on quality of life^62,63^ has led to suggestions that dysfunction in arousal and anxiety-related attentional processes could provide a useful biomarker for early identification of PwP who might develop FOG.^8,33^ On the other hand, postural instability and gait dysfunction symptoms were found to be risk factors for experiencing anxiety.^64^ Therefore, it is our understanding that the association between anxiety and FOG is most likely bidirectional, where anxiety and FOG could be part of a vicious cycle where one exacerbates the other. It is our understanding that when coming to target FOG related anxiety, the first step is identifying whether anxiety is a problem. This could be done using generic anxiety scale such as the Parkinson's Anxiety scale (PAS), however, this is not FOG specific. Another possibility is asking people if they feel that anxiety makes their FOG worse, but this does not identify worries (i.e., ruminations) as a particular problem that needs addressing. As such, providing a cut-off for worries/rumination might have an added value in this situation. The cut-off score in the current analysis is based on association with FOG frequency across the sample and therefore does not indicate the point at which worries might be considered a particular issue. However, it provides a step in this intended direction. Future studies should explore the potential to modify gait specific anxiety through interventions targeting the emotional and/or cognitive reaction of scenarios perceived to threaten balance and gait function (e.g., a supermarket entrance where FOG is often problematic).

This purported bidirectional association between FOG and anxiety suggests that multi-modal interventions may be required for treating/relieving FOG-related anxiety. On the one hand, targeting the cognitive process associated with ruminations and anxiety to reduce their effect on freezing, while on the other hand simultaneously targeting FOG and gait issues (which may also then reduce anxiety and ruminative thoughts).

With regard to the former, managing anxiety and associated worrisome thoughts may be a fruitful avenue for alleviating some of the motor symptoms associated with Parkinson's. However, effective strategies for managing anxiety and its influence on motor symptoms are currently insufficient.^65^ Typically, treatment approaches are pharmacological, involving drugs like selective serotonin reuptake inhibitors, benzodiazepines and antidepressant drugs^62,66^; and non-pharmacological, including cognitive behavioral therapy, transcranial magnetic stimulation, yoga and breathing control, to name a few.^66,67^ While a range of interventions have been presented, current evidence reporting the effectiveness of existing treatment options is weak.^62,68–70^ In a recent paper, Hinkle et al.,^71^ found a weak association between improved anxiety scores and change in motor symptoms in response to dopamine intake. Further study is warranted to decipher the neurophysiological and neurobiological associations between dysfunction in brain networks associated with arousal and cognition. However, since we don’t fully understand how anxiety effects PwP and its relation to FOG it is essential that the lived experiences of anxieties relating to FOG are considered within these endeavors and used in the design of non-pharmacological treatments. Ultimately, it would be highly valuable if this could lead to interventions and/or resources that help PwP to self-manage anxiety. With regard to managing FOG and other motor symptoms, Parkinson's medication such as levodopa has been found to only have a limited effect in anxiety subtype FOG.^72^ Therefore, other interventions for treating FOG may need to be further tested, including behavioral treatment, biofeedback and cueing, and surgical treatment including adaptive deep brain and spinal cord stimulation.^73^ This approach of treating FOG and its comorbidities is also supported by the literature and might be a promising direction for future interventions.^73^

### Limitations

The current study has several limitations. First, all measures were self-reported, and we therefore could not independently verify participants’ FOG status. However, the current approach did allow us to recruit a very large sample of PwP from across the UK, which is essential to perform a comprehensive scale validation. Nonetheless, it is possible that participants may have misunderstood certain questions or provided biased answers, given previous evidence of inaccuracies in self-reported levels of physical activity^74^ and FOG frequency.^75,76^ Regarding self-reporting of freezing frequency, we had decided not to restrict this question in time, based on interactions with our patient advisory group. However, this does mean that participants may have interpreted this question differently, e.g., due to differences in time since diagnosis. Nevertheless, the pattern of results presented here fits with previous reports (e.g., associations between freezing status and frequency with balance problems, years since diagnosis, etc.), and there are no clear additional sources of bias within our protocol that might compromise self-reported outcomes presented here.^77^

As our study is cross-sectional in nature, inferences regarding potential *causal* links between adapted G-SAP subscales and FOG cannot be made and require further study.

### Conclusion

The adapted G-SAP questionnaire could be used to monitor attentional constructs related to FOG in PwP. Our data suggests that perceptions of physiological arousal are associated with potentially more adaptive CMP in PwP-FOG. Conversely, PwP + FOG appear to demonstrate anxiety-related vulnerabilities characterized by a relative inability to engage in compensatory goal-directed focus of attention, potentially driven by heightened stimulus-irrelevant worrisome thoughts and associated worrisome thoughts. However, as was discussed above, it's also possible that previous experiences as well as deficits in cognition and associated top-down control may lead to increased self-reported ruminations in in that group. It is important to consider that the relationships between physiological arousal, CMP, and rumination with FOG frequency were observed when controlling for disease duration and self-reported balance impairment in the ordinal regression. While previous reports of higher G-SAP sub-scale scores in PwP + FOG were interpreted as potentially being driven by longer disease duration,^39^ the current data indicate that the relationship between physiological arousal and ruminations in PwP with frequent freezing could be driven, at least in part, by worrisome thoughts exacerbating FOG. Future research should investigate the interplay between worrisome thoughts and FOG. After all, tendencies in the way people allocate attention prior to or during FOG are perhaps more readily modifiable compared to progressive and chronic changes in automaticity/attentional capacity. Further study is required to test these ideas. Finally, we recommend that the current observations should be regarded as a starting point to consider the potential importance of G-SAP constructs in the context of FOG. Further work is necessary to describe the influence of deficits in cognition as well as more fundamental difficulties in both interoception^78^ and perceiving and describing emotions (alexithymia) on the relationships reported here. Indeed, we speculate that the adapted G-SAP subscales of arousal and rumination are likely to be particularly susceptible to deficits in introspection and alexithymia, respectively.

However, we also argue that the gait-specific nature of the G-SAP goes some way to overcoming the ambiguities of more generic scales evaluating thought-processes relating to movement in general (e.g., MSRS) and also consider it important that the adapted G-SAP does not specifically refer to experiences during FOG, but rather when walking in general.

## Supplemental Material

sj-docx-1-pkn-10.1177_1877718X251326266 - Supplemental material for Anxiety-related attentional characteristics and their relation to freezing of gait in people with Parkinson's: Cross-validation of the Adapted Gait Specific Attentional Profile (G-SAP) Supplemental material, sj-docx-1-pkn-10.1177_1877718X251326266 for Anxiety-related attentional characteristics and their relation to freezing of gait in people with Parkinson's: Cross-validation of the Adapted Gait Specific Attentional Profile (G-SAP) by Uri Rosenblum, Adam J Cocks, Meriel Norris, Elmar Kal and William R Young in Journal of Parkinson's Disease

sj-docx-2-pkn-10.1177_1877718X251326266 - Supplemental material for Anxiety-related attentional characteristics and their relation to freezing of gait in people with Parkinson's: Cross-validation of the Adapted Gait Specific Attentional Profile (G-SAP) Supplemental material, sj-docx-2-pkn-10.1177_1877718X251326266 for Anxiety-related attentional characteristics and their relation to freezing of gait in people with Parkinson's: Cross-validation of the Adapted Gait Specific Attentional Profile (G-SAP) by Uri Rosenblum, Adam J Cocks, Meriel Norris, Elmar Kal and William R Young in Journal of Parkinson's Disease
