# Supplementary material for: Anxiety-related attentional characteristics and their relation to freezing of gait in people with Parkinson's: Cross-validation of the Adapted Gait Specific Attentional Profile (G-SAP)
Source: J Parkinsons Dis. 2025 May 20;15(4):829–42. doi: 10.1177/1877718X251326266 (PMC13347471; doi:10.1177/1877718X251326266)
Supplement: sj-docx-1-pkn-10.1177_1877718X251326266 - Supplemental material for Anxiety-related attentional characteristics and their relation to freezing of gait in people with Parkinson's: Cross-validation of the Adapted Gait Specific Attentional Profile (G-SAP) [file sj-docx-1-pkn-10.1177_1877718X251326266.docx]

**Methods:**

**Adapted Gait-Specific Attentional Profile**

Date……………………………

VERY MUCH SO

OFTEN

MODERATELY SO

NOT VERY MUCH

NOT AT ALL

**Mark the appropriate circle to indicate**

**how you feel when you walk**

1 2 3 4 5

1 2 3 4 5

1 2 3 4 5

1 2 3 4 5

1 2 3 4 5

1 2 3 4 5

1 2 3 4 5

1 2 3 4 5

1 2 3 4 5

1 2 3 4 5

A1. I feel strained…………………………………………………………………………….

A3. I think about previous occasions when I lost my balance…………..

A4. I think about what would happen if I fell…………………………………..

A5. I get confused and make illogical decisions………….......................

A6. Worrisome thoughts about falling run through my mind………….

A7. I try to think about the way I walk/move……………........................

A8. I consciously try to control my movements……………………………….

A9. I examine the way I walk/move…………………………………………………

A10. I feel tense………………………………………………………………………………

A11. I find it difficult to concentrate on two things at once……………..

**To be completed by the researcher/clinician**

**Calculate the total score from each item relating to the four categories:**

Physiological arousal (sum of A1, A10) = ………………

Conscious movement processing (sum of A7, A8, A9) = ………………

Task-irrelevant ruminations/thoughts (sum of A3, A4, A6) = ………………

Processing inefficiencies (sum of A5, A11) = ………………

**Results:**

| **Supplemental Table 1.** Results of measurement invariance testing. | | | | | | | | |  |  |
| --- | --- | --- | --- | --- | --- | --- | --- | --- | --- | --- |
| **Invariance test** | **χ^2^** | **CFI**  **GFI** | **RMSEA** **(90%CI)** | **SRMR** | **Model comp.** | **∆χ^2^** | **∆CFI**  **∆GFI** | **∆RMSEA**  **∆SRMR** | | **Decision** |
| **1. Config.** | 123.330 df=58  *p*<0.001 | 0.965  0.946 | 0.051  [0.039, 0.064] | 0.043 | N/A | N/A | N/A | N/A | | Accept |
| **2. Metric** | 126.262 df=64  *p*<0.001 | 0.967  0.945 | 0.048  [0.035, 0.060] | 0.044 | 1 | 2.932 df=6  *p*=0.817 | 0.002  -0.001 | -0.003  0.001 | | Accept |
| **3. Scalar** | 160.288  df=76  *p*<0.001 | 0.954  0.931 | 0.052  [0.041, 0.063] | 0.060 | 2 | **34.025 df=10**  ***p*<0.001** | -0.013  -0.014 | 0.004  **0.016** | | (Accept)* |
| **3a. Partial** **Scalar**** | 149.564  df=73  p<0.001 | 0.959 0.935 | 0.050 [0.038, 0.061] | 0.054 | 3 | **23.302**  **df=9**  ***p*=0.006** | -0.008 -0.010 | 0.002  0.011 | | (Accept)* |
| **3b. Partial Scalar***** | 142.852  df=72  p<0.001 | 0.962 0.937 | 0.048 [0.036, 0.059] | 0.055 | 3a | **16.590** **df=8** ***p*=0.035** | -0.005 -0.008 | 0.000 0.012 | | (Accept)* |
| **NB:** CFI = Comparative fit index; Config. = Configural; GFI = Goodness-of-fit index; Model comp. = Model comparison; N/A= Not applicable; RMSEA = Root mean square error of approximation; SRMR = Standardized root mean squared residual; df = degrees of freedom; Model fit indices that exceed the threshold for acceptable model fit change are emphasized; * Scalar invariance was partly confirmed: ∆CFI, ∆GFI, ∆RMSEA were acceptable, but ∆χ^2^ was significant and ∆SRMR>0.015; ** Backward releasing of constraints revealed that allowing the covariance for ‘Physiological Arousal’ and ‘Conscious Movement Processing’ (model 3a) to differ across groups resulted in improved fit across all indices, except that ∆χ^2^ remained significant; *** Additional releasing of constraints related to the variance of scores on the Conscious Movement Processing subscale (model 3b) resulted in further significantly improved fit across all indices. While ∆χ^2^ remained significant, releasing of other constraints did not significantly improve model fit further. | | | | | | | | | | |


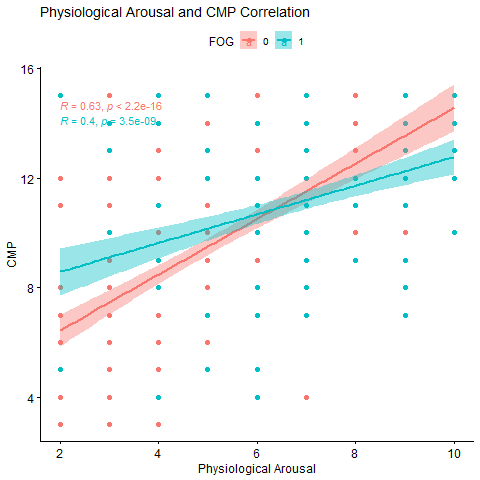


**Supplemental Figure 1.** Pearson correlation between Physiological arousal and conscious movement processing (CMP) across groups of people without freezing (red dots for raw data and red regression line with 95% confidence interval shaded in red) and of people with freezing (green dots for raw data and green regression line with 95% confidence interval shaded in green).


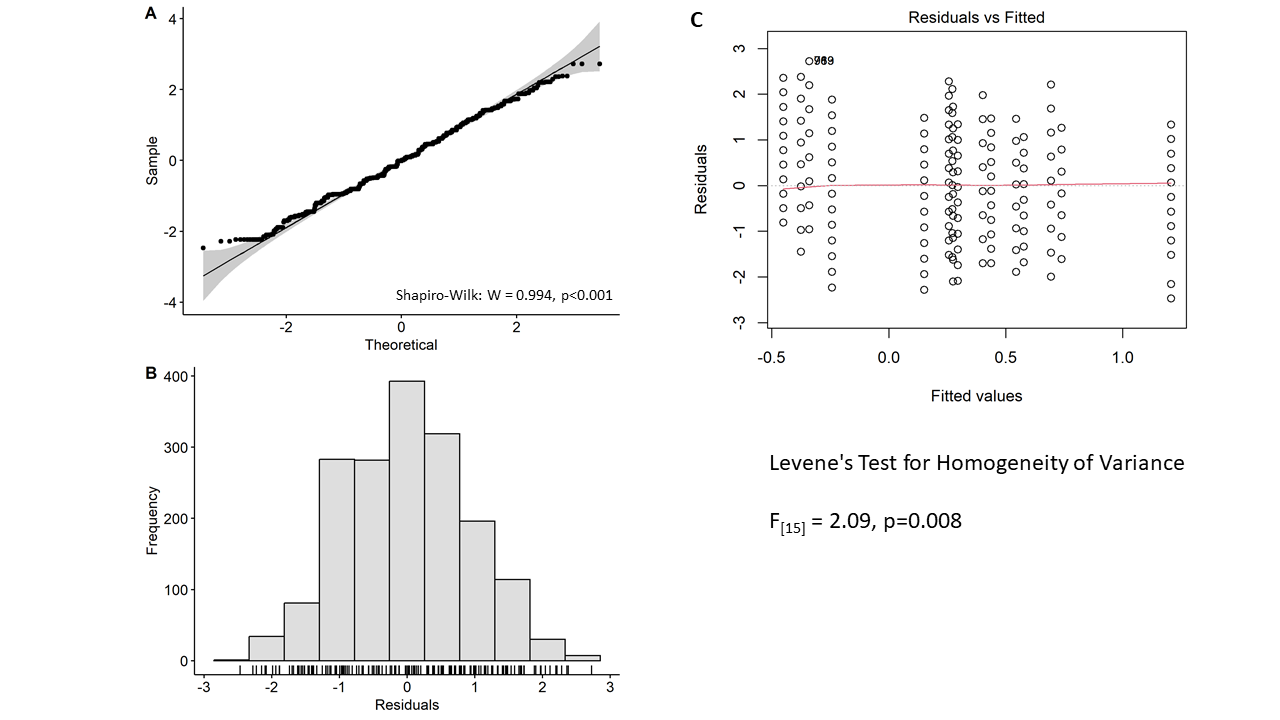


**Supplemental Figure 2.** Summery of assumptions testing for 2-way ANOVA where (A) is a Q-Q plot of ANOVA residuals (black dots) in relation to the [identity line](https://en.wikipedia.org/wiki/Identity_line) (y = x, black solid line) and their 95% confidence interval in shaded grey; (B) histogram of the residuals; and (C) Scatter plot of the residuals against the fitted values with a regression line (red solid line) for assessing homogeneity of variances.

***Adapted G-SAP scores: Association with frequency of freezing***

Multinomial logit model is summarized in Supplemental Table 2. The analysis revealed that all levels of frequency of freezing were significantly associated with years since diagnosis (Wald χ2 = 5.393-26.217, p’s≤0.020) and Rumination (Wald χ2 = 6.859-35.535, p’s≤0.009), such that the odds for a one-unit increase in the variable years since diagnosis is 1.084 for being in Hardly ever group vs. the Never group, 1.208 for being in Most weeks group vs. the Never, and 1.238 for being in the Everyday group vs. the Never group. The relative risk for a one-unit increase in the variable rumination is 1.218 for being in Hardly ever group vs. the Never group, 1.241 for being in Most weeks group vs. the Never, and 1.797 for being in the Everyday group vs. the Never group.

The Hardly ever group also showed significant associations with Age in years and Processing Inefficiency (Wald χ2 = 6.948, p=0.008, Wald χ2 = 4.267, p=0.039, respectively), such that the odds of being a year older is 0.959 and the relative risk for scoring one point higher on the Processing Inefficiency subscale is 1.192 for being in the Hardly ever group vs. the Never group. The Most weeks group also showed significant association with Physiological arousal (Wald χ2 = 9.458, p=0.002) such that the relative risk for a one-unit increase in the variable Physiological arousal is 1.543 for being in Most weeks group vs. the Never group. The Everyday group also showed significant association with Balance/gait problems (Wald χ2 = 3.893, p=0.049) such that the relative risk ratio for developing a Balance/gait problem is 0.102 for being in the Everyday group vs. the Never group.

| **Supplemental Table 2.** Results of multinomial logit model regression analysis of adapted G-SAP scores and freezing of gait frequency**.** | | | | |
| --- | --- | --- | --- | --- |
| **Freezing Frequency^a^** | | **OR [95% CI]** | **Wald χ^2^**  **(df=1)** | ***p*** |
|  |  |  |  |  |
|  |  |  |  |  |
| **Everyday** | Age in years | 1.042[0.990, 1.097] | 2.434 | .119 |
|  | Years since diagnosis | 1.238[1.141, 1.343] | 26.217 | **<.001** |
|  | Processing Inefficiency | 1.177[0.931, 1.486] | 1.859 | .173 |
|  | Physiological arousal | 1.208[0.919, 1.587] | 1.841 | .175 |
|  | Rumination | 1.797[1.482, 2.178] | 35.535 | **<.001** |
|  | Conscious Movement Processing | 1.016[0.825, 1.250] | 0.021 | .884 |
|  | Balance/gait problems^b^ | 0.102[0.011, 0.985] | 3.893 | **.049** |
| **Most weeks** | Age in years | 0.963[0.918, 1.010] | 2.460 | .117 |
|  | Years since diagnosis | 1.208[1.112, 1.313] | 19.790 | **<.001** |
|  | Processing Inefficiency | 1.196[0.945, 1.514] | 2.211 | .137 |
|  | Physiological arousal | 1.543[1.170, 2.035] | 9.458 | **.002** |
|  | Rumination | 1.241[1.056, 1.459] | 6.859 | **.009** |
|  | Conscious Movement Processing | 0.907[0.748, 1.099] | 0.995 | .319 |
|  | Balance/gait problems^b^ | 0.965[0.315, 2.963] | 0.004 | .951 |
| **Hardly ever** | Age in years | 0.959[0.929, 0.989] | 6.948 | **.008** |
|  | Years since diagnosis | 1.084[1.013, 1.161] | 5.393 | **.020** |
|  | Processing Inefficiency | 1.192[1.009, 1.409] | 4.267 | **.039** |
|  | Physiological arousal | 1.162[0.966, 1.399] | 2.529 | .112 |
|  | Rumination | 1.218[1.091, 1.360] | 12.310 | **<.001** |
|  | Conscious Movement Processing | 0.917[0.815, 1.030] | 2.129 | .145 |
|  | Balance/gait problems^b^ | 0.543[0.273, 1.082] | 3.011 | .083 |
| NB: OR = odds ratio, values>1 indicate increase in odds of experiencing more frequently freezing; df=degrees of freedom; Model-parameters: Improvement in fit vs. intercept-only model (*χ*^2^=235.335, df=21, *p*<0.001); Goodness-of-fit indices: Pearson (*χ*^2^=1193.434, df=1203, *p*=0.572), Deviance (*χ*^2^=686.858, df=1203, *p*=1.000); Nagelkerke pseudo R^2^=0.489.   1. The reference category is: Never. 2. Reference category is the self-reported problems with balance or gait group (N=293). | | | | |

***Adapted G-SAP scores cut off for predicting freezing***

| **Supplemental Table 3.** Measures of Diagnostic Accuracy from the Receiver Operating Characteristic (AUC) Curve Predicting FOG and freezing every day from G-SAP-PD, Rumination sub-scale. | | | | | |
| --- | --- | --- | --- | --- | --- |
|  | *cut-off score* | *Sensitivity* | *Specificity* | *Youden’s Index* | *AUC (95%CI)*  *P*  *Gini Index* |
| **PwP+FOG** |  |  |  |  | 0.777 (0.732-0.822)  <0.001  0.554 |
| Optional cut-off 1 | 5.5 | 0.838 | 0.543 | 0.381 |  |
| Optional cut-off 2 | **6.5** | **0.721** | **0.691** | **0.412** |  |
| Optional cut-off 3 | 7.5 | 0.609 | 0.787 | 0.396 |  |
| **PwP+FOG everyday** |  |  |  |  | 0.854 (0.794-0.915)  <0.001  0.709 |
| Optional cut-off 1 | 8.5 | .816 | 0.761 | .577 |  |
| Optional cut-off 2 | **9.5** | **.755** | **0.831** | **.586** |  |
| Optional cut-off 3 | 10.5 | .612 | 0.900 | .512 |  |

PwP+FOG = People with Parkinson’s that experience freezing of gait.
