# Supplementary material for: Anxiety-related attentional characteristics and their relation to freezing of gait in people with Parkinson's: Cross-validation of the Adapted Gait Specific Attentional Profile (G-SAP)
Source: J Parkinsons Dis. 2025 May 20;15(4):829–42. doi: 10.1177/1877718X251326266 (PMC13347471; doi:10.1177/1877718X251326266)
Supplement: sj-docx-2-pkn-10.1177_1877718X251326266 - Supplemental material for Anxiety-related attentional characteristics and their relation to freezing of gait in people with Parkinson's: Cross-validation of the Adapted Gait Specific Attentional Profile (G-SAP) [file sj-docx-2-pkn-10.1177_1877718X251326266.docx]

**Additional analysis including all three original items on the anxiety subscale of the GSAP:**
**We have re-run the main validity/reliability analyses, and ordinal regression analysis to show if and how results of these would change if we would have included the original Anxiety subscale as in the original GSAP validation study presented by Young et al. (2020) (as opposed to the renamed ‘Physiological Arousal’ subscale as in the main paper presented here). Results are presented below.**

1. Structural validity and measurement invariance

The overall CFA model now also **including the original anxiety question** is presented below. Medium to strong correlations were observed between all factors, and especially between the factor of ‘Anxiety’ and respectively ‘Conscious Movement Processing’ (r=0.72), Rumination (r=0.70), and Processing inefficiency (r=0.69). Standardised item-factor loadings were all positive and high (≥0.70). Model fit was highly similar to the model without the original anxiety item included (χ^2^_(38)_=86.782, *p*<0.001; χ^2^/df=2.284; CFI=0.981; GFI=0.965; RMSEA=0.054[0.039, 0.070]; SRMR=0.033).

The model demonstrated configural and metric measurement invariance (see Table R1 below). Scalar measurement invariance was borderline satisfactory. Further analysis using backward releasing of constraints revealed that this was primarily due to a significant between-group difference in covariance between the subscales ‘Anxiety and ‘CMP’ (*r*_PwP+FOG_=0.57, *r*_PwP-FOG_=0.78, respectively; *Z*=-2.659, *p*=0.011), and to a lesser extent to reduced variability in ‘CMP’ subscale scores among PwP+FOG compared to PwP-FOG (6.40 and 9.25, respectively; *Z*=-2.200, p=0.028). Unconstrained values for the ‘Anxiety’ and ‘CMP’ covariance, and ‘CMP’ variance led to acceptable partial scalar invariance (Table R1).

**Overall, the inclusion of the previously excluded ‘Anxiety’ item led to highly similar results for the CFA and measurement invariance testing compared to the results presented in the main text: Results overall confirmed the hypothesised four-factor structure of the adapted G-SAP, and that the scale is suitable to compare scores between PwP+FOG and PwP-FOG.**

1. *Internal consistency*

Internal consistency of the adapted G-SAP was confirmed. For the sample overall, standardized Cronbach’s alpha values were 0.79, 0.86, 0.89 and 0.67 for Physiological Arousal, CMP, Rumination and PI, respectively. For PwP+FOG the respective standardized Cronbach’s alpha values were 0.72, 0.82, 0.86, and 0.65, while for PwP-FOG these were 0.77, 0.87, 0.85, and 0.61.

**Overall, the inclusion of the previously excluded ‘Anxiety’ item led to highly similar results for the internal consistency results – alpha was somewhat higher, but not to the extent that this would have altered main conclusions of the paper. Also note that Cronbach’s alpha should be interpreted with care. For instance, alpha by default is significantly biased upwards as greater numbers of items are included, even if these additional items are only minimally associated to one another (see e.g. Cortina et al. (1993),** [**https://psycnet.apa.org/doi/10.1037/0021-9010.78.1.98**](https://psycnet.apa.org/doi/10.1037/0021-9010.78.1.98)**).**

1. *Adapted G-SAP scores - association with frequency of freezing*

Ordinal regression results are presented in Table R2. Twenty-seven participants with missing responses for age, years since diagnosis or experiencing balance problems were not included in the regression analysis. Only higher Rumination subscale scores (OR=1.326, 95% CI = [1.216, 1.446]) and higher Anxiety subscale scores (OR=1.121, 95% CI = [1.011, 1.243]) were associated with significantly greater odds of experiencing more frequent freezing. With regard to the control variables, years since diagnosis (OR=1.137, 95% CI = [1.088, 1.189]) and balance/gait problems were significantly associated with freezing frequency (OR=0.539, 95% CI = [0.298, 0.975]).

**Overall, the main ordinal regression results were highly similar to the results presented in the main paper. However, note that the odds ratios between ‘Anxiety’ and freezing frequency was markedly lower (~0.07) compared to the ‘Physiological Arousal’ subscale, suggesting that the removal of the ‘concerns’ item may have a positive effect on the utility of the subscale.**

| **Table R1.** Results of measurement invariance testing. | | | | | | | | | |  |
| --- | --- | --- | --- | --- | --- | --- | --- | --- | --- | --- |
| **Invariance test** | **χ^2^** | **CFI**  **GFI** | **RMSEA (90%CI)** | **SRMR** | **Model comp.** | **∆χ^2^** | **∆CFI**  **∆GFI** | **∆RMSEA**  **∆SRMR** | **Decision** | |
| **1. Config.** | 138.885 df=76  *p*<0.001 | 0.969  0.946 | 0.044  [0.032, 0.055] | 0.042 | N/A | N/A | N/A | N/A | Accept | |
| **2. Metric** | 142.731 df=83  *p*<0.001 | 0.971  0.944 | 0.041  [0.029, 0.052] | 0.044 | 1 | 3.864 df=7  *p*=0.797 | 0.002  -0.002 | -0.003  0.002 | Accept | |
| **3. Scalar** | 169.901  df=93  *p*<0.001 | 0.963  0.934 | 0.044  [0.033, 0.054] | 0.057 | 2 | **27.170 df=10**  ***p*=0.002** | -0.008  -0.010 | 0.003  0.013 | (Accept)* | |
| **3a Partial Scalar**** | 164.446  df=92  p<0.001 | 0.965 0.935 | 0.043 [0.032, 0.053] | 0.053 | 2 | **21.714**  **df=9**  ***p*=0.010** | -0.006 -0.009 | 0.002  0.009 | (Accept)* | |
| **3b Partial Scalar***** | 158.248  df=91  p<0.001 | 0.967 0.938 | 0.041 [0.030, 0.052] | 0.054 | 2 | 15.517 df=8 *p*=0.050 | -0.004 -0.006 | 0.000 0.010 | Accept | |
| **NB:** CFI = Comparative fit index; Config. = Configural; GFI = Goodness-of-fit index; Model comp. = Model comparison; N/A= Not applicable; RMSEA = Root mean square error of approximation; SRMR = Standardized root mean squared residual; df = degrees of freedom; Model fit indices that exceed the threshold for acceptable model fit change are emphasized; * Scalar invariance was partly confirmed: ∆CFI, ∆GFI, ∆RMSEA, and ∆SRMR were acceptable, but ∆χ^2^ was significant; ** Backward releasing of constraints revealed that allowing the covariance for ‘Anxiety’ and ‘Conscious Movement Processing’ (model 3a) to differ across groups resulted in improved fit across all indices, except that ∆χ^2^ remained significant; *** Additional releasing of constraints related to the variance of scores on the Conscious Movement Processing subscale (model 3b) resulted in further significantly improved fit across all indices. | | | | | | | | | | |
|  | | | | | | | | | | |

| **Table R2.** Results of ordinal regression analysis of adapted G-SAP scores as a function of freezing of gait frequency**.^a^** | | | |
| --- | --- | --- | --- |
|  | **OR [95% CI]** | **Wald χ^2^**  **(df=1)** | ***p*** |
| Age in years | 0.990 [0.966, 1.016] | 0.555 | 0.456 |
| Years since diagnosis | 1.137 [1.088, 1.189] | 32.313 | **<.001** |
| Processing Inefficiency | 1.129 [0.995, 1.280] | 3.566 | 0.059 |
| Anxiety | 1.121 [1.011, 1.243] | 4.702 | **0.030** |
| Rumination | 1.326 [1.216, 1.446] | 41.058 | **<.001** |
| Conscious Movement Processing | 0.957 [0.867, 1.056] | 0.773 | 0.379 |
| Balance/gait problems^b^ | 0.539 [0.298, 0.975] | 4.172 | **0.041** |
| NB: OR = odds ratio, values>1 indicate increase in odds of experiencing more frequently freezing; df=degrees of freedom; Model-parameters: Improvement in fit vs. intercept-only model (*χ*^2^=200.515, df=7, *p*<0.001); Goodness-of-fit indices: Pearson (*χ*^2^=1119.489, df=1217, *p*=0.978), Deviance (*χ*^2^=721.678, df=1217, *p*=1.000); Nagelkerke pseudo R^2^=0.433.   1. The assumption of lack of multicollinearity was met (all VIFs 1.096-2.342), but the proportional odds assumption was not, as evidenced by a significant test of parallel lines (χ2= 44.794, df=14, p<0.001). 2. Reference category is group with self-reported problems with balance or gait (N=293). | | | |
